# Morphological risk factors for scaphoid fracture: a case–control study

**DOI:** 10.1007/s00068-022-02101-y

**Published:** 2022-09-27

**Authors:** Abigael Cohen, Thomas Claessen, Corne van den Berg, Michiel Siebelt, Tjebbe Hagenaars, Gerald A. Kraan, Johannes H. Waarsing, Max Reijman, Joost W. Colaris

**Affiliations:** 1 Department of Orthopaedic Surgery and Sports Medicine, Erasmus MC University Medical Center, Room Nc-424, PO Box 2040, 3000 CA Rotterdam, The Netherlands; 2 Department of Neurology, Dijklander Hospital, Maelsonstraat 3, 1624 NP Hoorn, The Netherlands; 3 Department of Orthopaedic Surgery, St. Anna Hospital, Bogardeind 2, 5664 EH Geldrop, The Netherlands; 4 Trauma Research Unit, Department of Surgery, Erasmus MC University Medical Center Rotterdam, PO Box 2040, 3000 CA Rotterdam, The Netherlands; 5 Department of Emergency Medicine, Erasmus MC University Medical Center Rotterdam, PO Box 2040, 3000 CA Rotterdam, The Netherlands; 6 Department of Orthopaedic Surgery, Reinier HAGA Orthopaedic Center, Toneellaan 2, 2725 NA Zoetermeer, The Netherlands

**Keywords:** Wrist, Scaphoid bone, Risk factors, Adult, Humans, Fractures, Bone, Contusions

## Abstract

**Purpose:**

Most patients with a clinically suspected scaphoid fracture and normal initial radiograph are unnecessarily treated. Previously developed prediction rules using demographic and clinical risk are unable to accurately predict occult fractures. Adding other risk factors could enhance this. Therefore, we aim to explore if there are morphological risk factors of the wrist for sustaining a scaphoid fracture.

**Methods:**

We retrospectively included adult patients with a clinically suspected scaphoid fracture between 2013 and 2019 in our case–control study. There were 82 patients with a scaphoid fracture and 158 patients with a wrist contusion. Morphological risk factors were identified using statistical shape modelling (SSM) and linear measurements. Independent wrist shape variations on posteroanterior and lateral radiographs were captured in modes using SSM. Associations between outcomes and a scaphoid fracture were explored using logistic regression and the reliability was assessed.

**Results:**

Of the 15 posteroanterior modes and 8 lateral modes identified and linear measurements performed, 1 PA mode was associated with a scaphoid fracture (PA mode 4; OR 1.40, CI 1.04–1.93, *p* = 0.031). We described this mode as an ulna plus and narrower distal radius with more volar tilt and radial inclination. The reliability of the posteroanterior modes and linear measurements was mostly good/excellent and moderate/poor for the lateral modes.

**Conclusion:**

There was one complex wrist shape significantly associated with a scaphoid fracture. Since the association was weak and the shape is difficult to identify radiographs, we believe this morphological risk factor would not enhance identifying occult scaphoid fractures in the future.

**Supplementary Information:**

The online version contains supplementary material available at 10.1007/s00068-022-02101-y.

## Introduction

### Background

Ninety percent of the patients with a clinically suspected scaphoid fracture and normal initial radiographs are unnecessarily treated with cast for 2 weeks since only 10% have an occult scaphoid fracture [5]. This unnecessary cast treatment leads to avoidable healthcare costs and loss of productivity [23]. However, identifying scaphoid fractures is even challenging on imaging since they can be missed on initial radiographs and CT, and patients could be falsely diagnosed with a scaphoid fracture on MRI [27]. The fact that untreated scaphoid fractures can become a non-union leading to progressive wrist osteoarthritis, is the rationale behind treating patients with a clinically suspected scaphoid fracture and normal initial radiographs with cast [10, 14, 18]. Identifying high-risk patients for an occult scaphoid fracture at the emergency department could reduce unnecessary cast treatment. Previously published studies have developed prediction rules with low accuracy. The study by Rhemrev et al. [22] developed a prediction rule for scaphoid fractures with a sensitivity of 0.15 and specificity of 0.98, which included the presence of a previous fracture of the hand or wrist, wrist extension < 50%, and supination strength ≤ 10%. Wrist extension and supination strength are usually not measured at the emergency department since a Hand Dynamometer is needed to measure them. Duckworth et al. [6] developed another prediction rule for scaphoid fractures with a sensitivity of 0.82 and specificity of 0.80, including male gender, sports injury, anatomical snuffbox pain on ulnar deviation of the wrist, and thumb-index finger pinch [6]. Morphological risk factors are lacking in these prediction rules. If there is a high association between a scaphoid fracture and wrist morphology, adding morphology to a prediction rule could easily enhance them in the future to distinguish between high-risk and low-risk patients for an occult scaphoid fracture. Recently, a study by Turan et al. [28] reported an association between a scaphoid fracture and a wrist with increased radial inclination, volar tilt, radial height, and ulna minus variant after fall onto outstretched hand (FOOSH). Due to the complex shape of the wrist, it is challenging to capture all shape variants with linear measurements accurately. In other joints, statistical shape modeling (SSM) is used to explore shape variations [1, 3]. Therefore, we aim to explore if shape variations of the distal radius and ulna are associated with sustaining a scaphoid fracture after FOOSH using statistical shape modeling and linear measurements.

## Patients and methods

### Study design and setting

We collected data to perform this case–control study at two hospitals in the Netherlands (Reinier de Graaf Gasthuis and Erasmus MC University Medical Center). The study is written following the Strengthening The Reporting of Observational studies in Epidemiology (STROBE) statement [30]. The study was performed following the declaration of Helsinki and approved by the local medical ethical research committee (MEC-2019-0026).

### Participants

Adult patients (≥ 18 years) who presented at the emergency department with a clinically suspected scaphoid fracture after a FOOSH were eligible for this study. A scaphoid fracture was clinically suspected if patients reported radial-sided wrist pain after trauma and the scaphoid was tender at palpation. Patients were excluded if they had; (1) a previous fracture of the radius or ulna, (2) congenital disease or abnormality of the arm, (3) other diagnoses than scaphoid fracture or wrist contusion during follow-up, (4) no adequate follow-up to set the diagnosis scaphoid fracture or wrist contusion, (5) unavailable posteroanterior (PA) or lateral (LA) wrist radiograph, or (6) quality wrist radiograph unsuitable for SSM.

The electronic patient record was screened from 01-01-2013 to 01-01-2019, which made it possible that minimally 1-year follow-up data were available. At the Erasmus MC University Medical Center, all patients with radiographs of the scaphoid were screened. At the Reinier de Graaf Gasthuis, all adult patients who presented at the emergency department between the screening dates with synonyms of “scaphoid” in their electronic patient record were screened. We collected data from the electronic patient record regarding baseline demographics (e.g., age and gender), mechanism of injury (e.g. low energetic trauma, high energetic trauma, sports injury), final diagnosis (scaphoid fracture or wrist contusion), and imaging.

Due to the fact that there is no gold standard for diagnosing a scaphoid fracture, we used previously reported definitions for the presence or absence of a scaphoid fracture [6, 15]. Included patients were allocated to the case group (scaphoid fracture group) if a scaphoid fracture was radiographically confirmed during 6 weeks’ follow-up (CT, MRI, or radiograph) by the radiologist and physician. Patients were allocated to the control group (wrist contusion group) if there was no scaphoid tenderness within 6 weeks after the trauma and/or the radiologist reported no scaphoid fracture on imaging during 6 weeks’ follow-up (CT, MRI, or radiograph) and there was no scaphoid non-union reported in the electronic patient record. We included patients with at least 1 year of follow-up to evaluate if patients returned to the hospital due to a scaphoid non-union.

### Study population

**Fig. 1:**
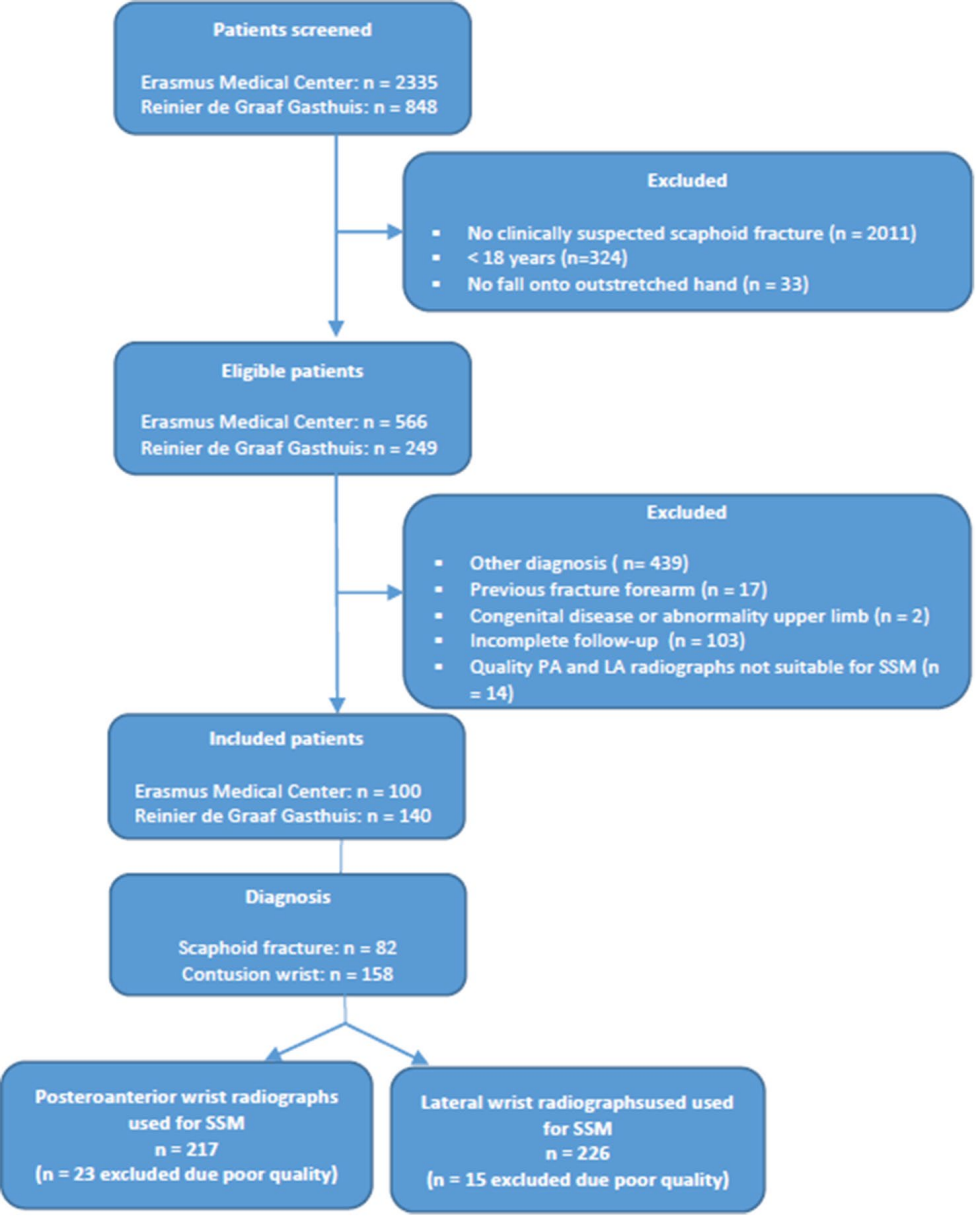
Study flowchart

After applying our inclusion and exclusion criteria, we included 240 consecutive patients in our study. Of these patients, 82 were allocated to the fracture group and 158 to the contusion group. A total of 217 PA and 226 LA wrist radiographs were available for SSM (Fig. 1).

Patients with a scaphoid fracture were mainly male (*p* = 0.007) and younger (*p* = 0.003) than patients in the wrist contusion group (Table 1). The physician diagnosed patients with a scaphoid fracture mostly based on radiographs (*n* = 74) and less often on CT (*n* = 7) or MRI (*n* = 1). Patients were diagnosed with a contusion of the wrist after either a normal CT (*n* = 20), normal MRI (*n* = 4), normal bone scintigraphy (*n* = 1), normal radiographs after 6 weeks (*n* = 4) or no pain during physical examination within 6 weeks follow-up with normal radiographs (*n* = 129).

**Table 1:** Baseline characteristics

	Fracture group (*N* = 82)	Contusion group (*N* = 158)
Age, median [IQR]	27 [22-44]	39 [24–57]
Male sex, % (*N*)	76 (62)	57 (90)
Trauma, % (*N*)		
Fight	2 (2)	2 (3)
Sports injury	12 (10)	8 (13)
Low energetic trauma	80 (66)	85 (135)
High energetic trauma	5 (4)	4 (7)

*IQR* inter quartile range

### Statistical shape modeling

We assessed the association between a shape variation and sustaining a scaphoid fracture using SSM. The radiographs used to construct PA and LA statistical shape models were made according to a standard protocol. PA wrist radiographs were made with the patient’s shoulder in 90 degrees of abduction, elbow in 90 degrees of flexion, the wrist in pronation, and fingers slightly flexed. The elbow remained in 90 degrees flexion for the LA radiographs, however, the shoulder and wrist were in a neutral position with the radial and ulnar styloid processes precisely on top of each other. The tube was centered on the wrist joint for both radiographs.

**Fig. 2:**
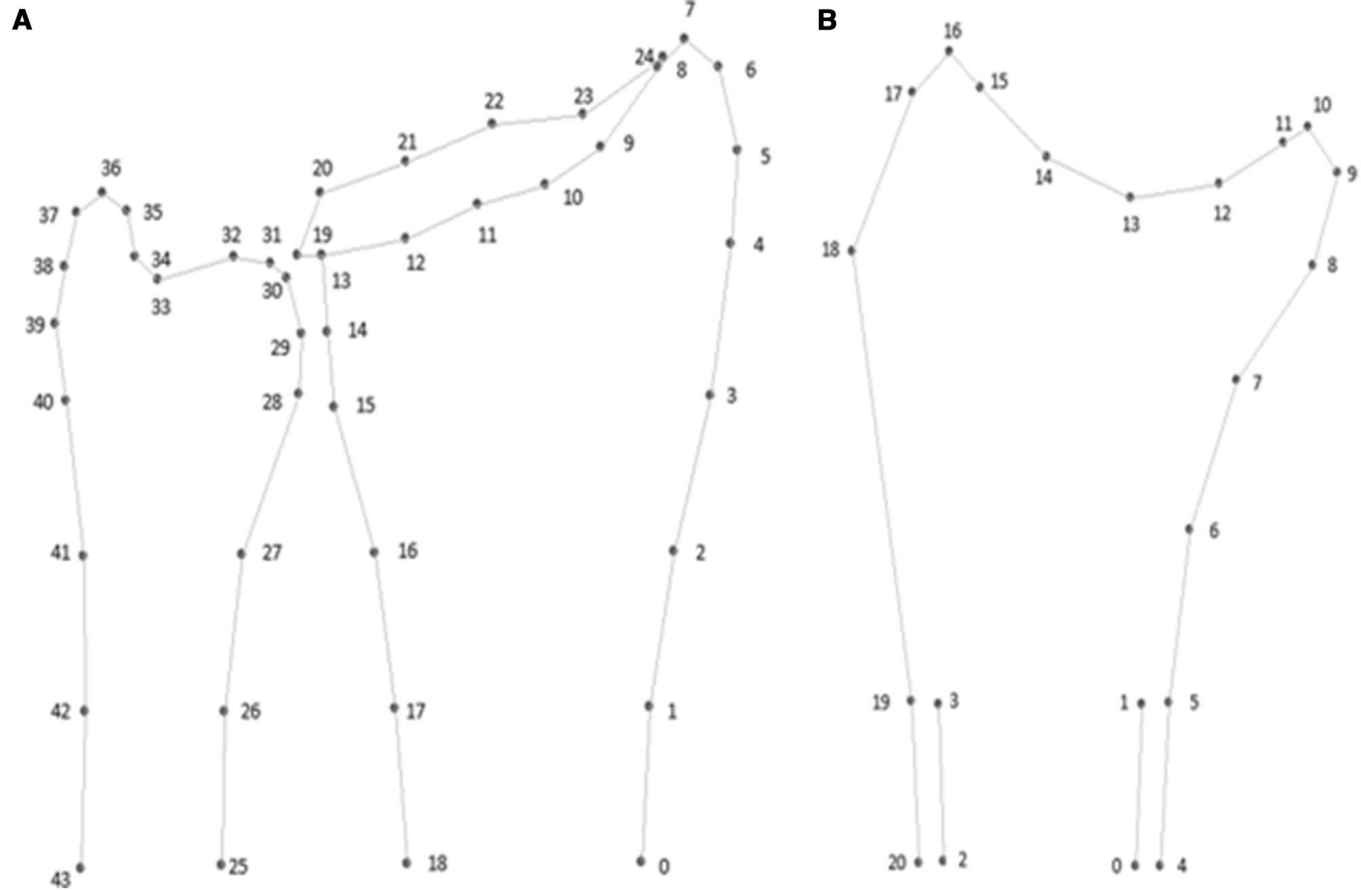
Point set of statistical shape model. **A** 44 point set of the statistical shape model of the posteroanterior wrist radiograph. **B** 21 point set of the statistical shape of the lateral wrist radiograph

To quantify shape variants of the distal radius and ulna within our population, we manually placed points along the bone contour of the distal radius and ulna on radiographs using SSM Software (ASM tool kit of the Manchester University) [11]. A protocol with 44 points along the outline of the distal radius and ulna on PA radiographs and 21 points on LA radiographs was made to ensure that each landmark was placed on the same anatomical landmark (Fig. 2). After all, the points were placed on the radiograph using SSM software, the individual points of each radiograph were checked and adjusted when necessary using Bonefinder [12]. After that, a PA and LA statistical shape model was constructed using the ASM tool kit. We extracted numerous independent SSM modes, calculated without a hypothesis, that describe 90% shape variations of the distal radius and ulna within our study population. Each mode represents an independent shape variant. For each mode, the mean shape of our study population is quantitatively described as zero. A positive or negative deviation from the mean of each mode is calculated per patient using Matlab V.7.1.0 (MathWorks, Natick, Massachusetts, USA). To standardize the outcome, we expressed the positive or negative deviation as the standard deviation from the mean shape of each mode per patient.

### Linear measurements

**Fig. 3:**
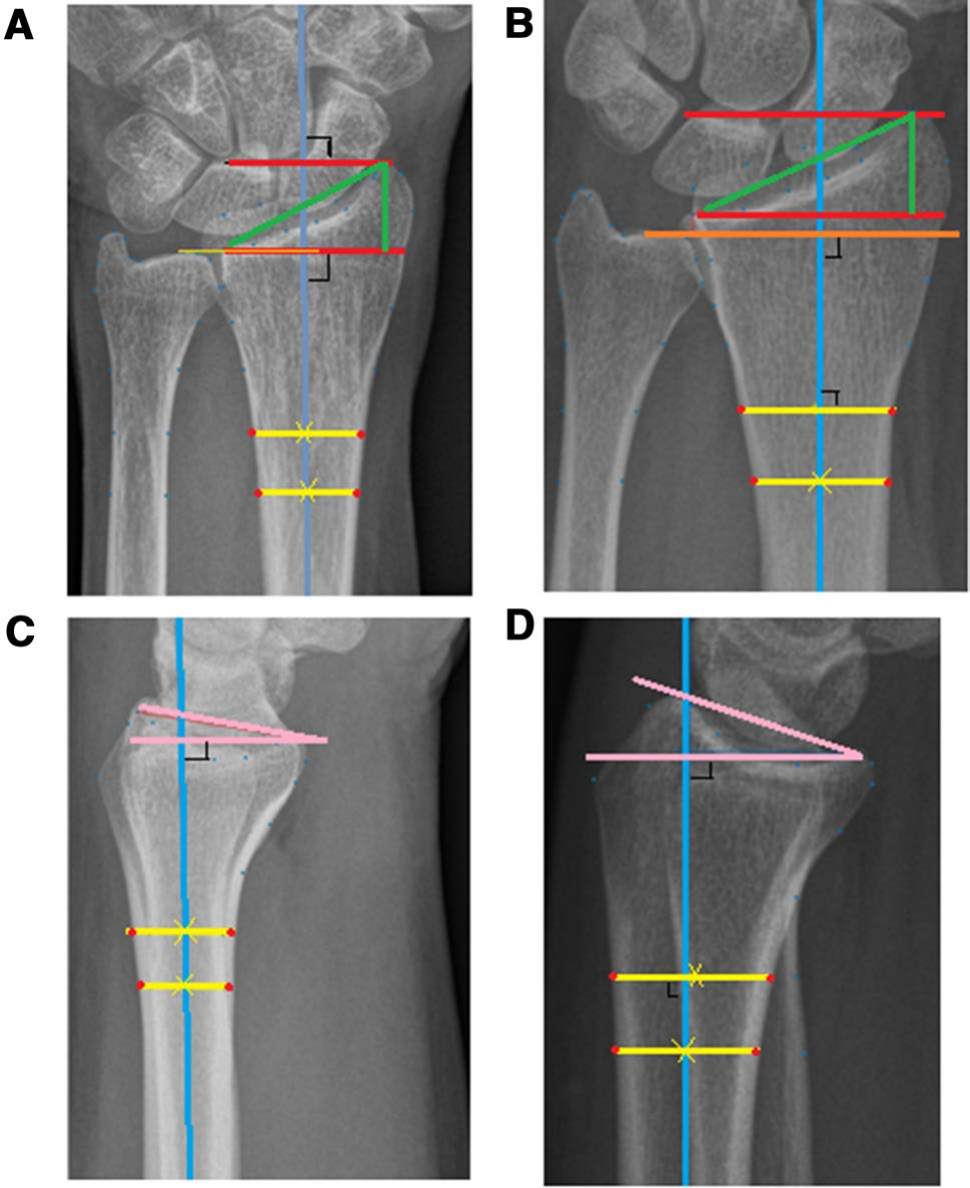
Linear measurements. **A**, **C** Perpendicular line to the long axis of the radius is drawn through the middle of the two yellow lines or **B**, **D** through the middle of the proximal yellow line and perpendicular to the distal yellow line. **A**, **B** Posteroanterior wrist radiographs with measurements of (1) Ulnar variance: the orange line, which is touching the distal cortical rim of the ulna, is proximal (minus) or distal (plus) to the red line which is touching the distal ulnar aspect of the radius. (2) Relative radial height: The length of the proximal red line divided by the length of the vertical green line which is touching the tip of the radial styloid. (3) Radial inclination: The angle between green line from the distal ulnar aspect of the radius to the tip of the radial styloid and the proximal red line. **C**, **D** Lateral wrist radiographs with measurements of volar tilt: The angle between the pink lines which is touching the most distal points of the volar and dorsal ridge of the radius

The association between linear measurements such as radial inclination, volar tilt, relative radial height, and ulnar variance (Fig. 3) and sustaining a scaphoid fracture was assessed in this study. We used a similar method as previously described to measure the alpha angle in the hip to define cam morphology [29].

Landmarks necessary to perform linear measurements were included in the protocol to create our statistical shape model. These measurements were calculated using Matlab V.7.1.0 (MathWorks, Natick, Massachusetts, USA). The perpendicular line to the long axis of the radius is drawn through either the middle of the two lines on the radial shaft or through the middle of the proximal radial shaft line and perpendicular to the distal radial shaft line. The perpendicular line to the long axis of the radius that represented the radius shaft center best was used for the measurements (Fig. 3). Since linear measurements were done on radiographs that were not calibrated, we measured relative radial height instead of radial height, and expressed ulnar variance only as ulna minus or ulna plus.

### Reliability

To evaluate the intra-observer reliability of SSM and linear measurements, the same observer placed points along the outline of the distal radius and ulna on 20 randomly chosen PA and LA wrist radiographs 1 month after the initial statistical shape models were made. A second researcher placed points on the same randomly chosen 40 radiographs to evaluate the inter-observer variability.

### Statistical analysis

We tested the distribution of baseline variables by the Shapiro–Wilk test. The mean with ranges for normally distributed variables were reported, and the median with interquartile ranges (IQR) for not normally distributed variables. We performed an univariate logistic regression analysis to assess whether there was an association between the presence and absence of a scaphoid fracture (dependent variable) and shape variation (SSM mode) or linear measurement (independent variables). We adjusted the univariate logistic regression model for age and gender since these characteristics could influence wrist shape. Odds ratios (OR), 95% confidence intervals (95% CI), and *p* values were calculated for each mode and each linear measurement. Since this was an explorative study, we did not correct for multiple testing.

The inter-observer and intra-observer reliability were computed by calculating the intra-class correlation coefficient (ICC) using a two-way random-effect model with absolute agreement. We interpreted the ICC as follows; ICC < 0.50 (poor agreement), ICC 0.50–0.75 (moderate agreement), value 0.75–0.90 (good agreement), value > 0.90 (excellent agreement) [19].

We used R statistical computing, version 1.2.5001, for all analyses. A *p* value smaller than 0.05 was considered statistically significant.

## Results

### Identifying morphological risk factors

**Fig. 4:**
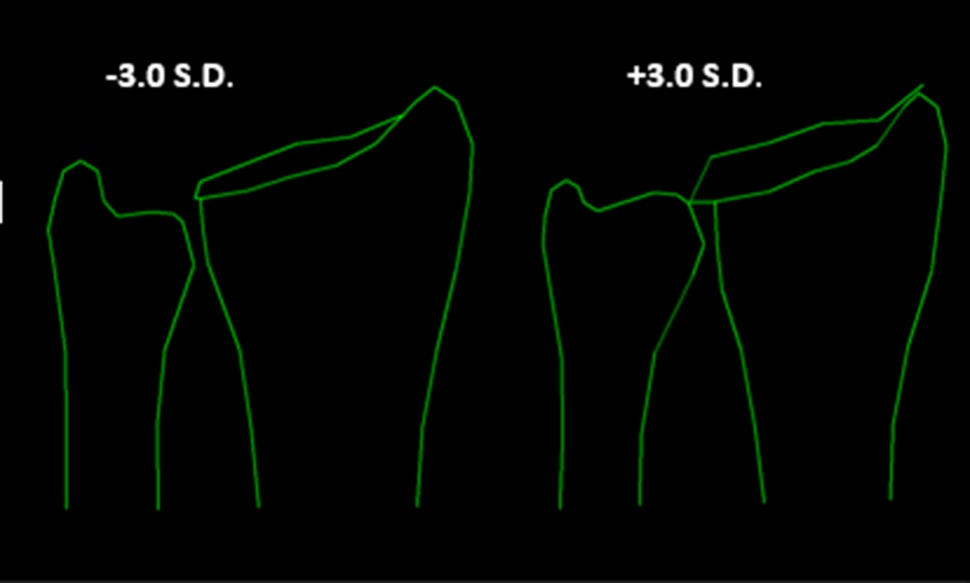
Posteroanterior (PA) mode 4 based on PA radiographs; On the left − 3.0 standard deviation (S.D.) from the mean mode and on the right the 3.0 S.D. from the mean mode. Higher values were associated with a scaphoid fracture

We found 15 PA modes and 8 LA modes that together explained 90% of the distal radius and ulna shape variances on the PA radiographs and LA radiographs within our study population (Supplementary Table 1). Only mode 4 of the PA shape variants were associated with a scaphoid fracture (OR 1.40 (95% CI 1.04–1.93, *p*-value = 0.031). The shape of the distal radius and ulna represented by PA mode 4 shows an association between a scaphoid fracture and ulna plus with a narrower distal radius with more volar tilt and more radial inclination (Fig. 4) compared to patients with a wrist contusion. No association was found between the LA shape variants and a scaphoid fracture. Using linear measurements, we found no association between sustaining a scaphoid fracture and ulnar variance, relative radial height, radial inclination, or volar tilt (Table 2).

## Inter-observer and intra-observer reliability

The inter-observer and intra-observer reliability of SSM of PA mode 4 was good, with an intra-class correlation coefficient of respectively 0.90 (95% CI 0.77 to 0.96) and 0.89 (95% CI 0.73 to 0.96). For the other PA shape variations, the inter-observer reliability was good or excellent in 86% of the modes, and the intra-observer reliability was good or excellent in 93% of the modes. On the contrary, the reliability for the LA shape variations was mostly moderate or poor, with only one mode with good inter-observer reliability and three modes with either excellent or good intra-observer reliability (Supplementary Table 1). The reliability of the linear measurements performed in our study was mainly excellent or good (Table 2).

**Table 2:** Association linear measurements and scaphoid fracture

	Adjusted odds ratio (95% CI)	*p*-value	Inter-observer ICC (95% CI)	Intra-observer ICC (95% CI)
Ulnar variance ( +)	1.03 (0.57–1.85)	0.933		
Relative radial height	2.11 (0.008–613.2)	0.795	0.77 (0.52–0.90)	0.95 (0.87–0.98)
Radial inclination	1.00 (0.90–1.11)	0.977	0.71 (0.41–0.87)	0.95 (0.87–0.98)
Volar tilt	1.01 (0.95–1.08)	0.695	0.93 (0.83–0.97)	0.91 (0.79–0.96)

The odds ratio is adjusted for age and gender

The intra-class correlation coefficient (ICC) with the 95% confidence interval (CI) is reported

## Discussion

Most patients (90%) with a clinically suspected scaphoid fracture and normal initial radiographs are unnecessarily treated with cast to prevent untreated scaphoid fractures from becoming nonunions that lead to wrist osteoarthritis [5]. This overtreatment could be minimized if we could adequately identify patients with an occult scaphoid fracture. Previously developed prediction rules to identify occult scaphoid fractures with demographic and/or clinical findings are not accurate [6, 22], and adding different risk factors could enhance this in the future. After exploring morphological risk factors for a scaphoid fracture using statistical shape modeling and linear measurements on wrist radiographs, we found a weak association between one complex shape variation of the distal radius and ulna on posteroanterior (PA) radiographs and a scaphoid fracture after FOOSH. Thereby, the shape variations identified with SSM on PA radiographs and the linear measurements were reliable, but the reliability of the lateral shape variations with SSM was moderate to poor. However, the morphological risk factor we identified is challenging to observe for a physician on a radiograph at the emergency department. Furthermore, the association is weak and therefore morphology of the radius and ulna might play a small role in sustaining a scaphoid fracture. We believe including this shape variation into a prediction rule in the future with demographic and/or clinical risk factors would not enhance identifying high-risk patients for an occult scaphoid fracture.

We described the shape variation that was associated with a scaphoid fracture after fall onto outstretched hand (FOOSH) as an ulna plus with a narrower distal radius with more volar tilt and higher radial inclination compared to patients with a wrist contusion. Weber et al. [31] reported that scaphoid waist fractures occur mainly during 95–100 degrees of extension. A fall onto an outstretched hand results in extreme extension of the wrist, a vertically orientation of the scaphoid with the stabilized proximal pole of the scaphoid between the dorsal ridge of the radius, the volar ligaments, and the capitate [13, 31]. The force transmission in the radiocarpal joint shifts dorso-radially with high-pressure areas concentrated at the distal and proximal parts of the scaphoid with more tension in the volar ligaments [13, 16, 20]. An external force from the trapezium to the distal pole of the scaphoid can cause a scaphoid fracture due to bending force [13]. Therefore, the risk of fracturing the scaphoid may also depend on the point of load application on the palm during a fall (ulnar or radial deviation) and if there is a previous ligamental injury [4, 13].

We hypothesize that the most morphological features of this shape variation resulted in higher force transmission through the scaphoid during a FOOSH which causes a scaphoid fracture. First, a narrower distal radius and increased radial inclination will result in higher pressure per cm^2^ [11]. Second, due to the higher dorsal ridge of the radius in patients with more volar tilt, the scaphoid waist will bend over the dorsal ridge causing a scaphoid fracture. Third, in contrary, an ulna plus is associated with an increase in load at the ulnocarpal joint as seen in patients with an ulna impaction syndrome. This is seen due arthritic changes at the dome of the ulna, the proximal ulnar corner of the lunate or the proximal radial corner of the triquetrum in patients with the ulna impaction syndrome. In such cases, an ulna shorting results in a significant decrease in ulnar load [24]. However, though the ulna is the most positive during FOOSH due to pronation of the forearm [2], the force transmitted to the ulnar column is less than expected due a volar-dorsal shift in the position of the ulnar head relative to the carpus [17, 26]. In patients with an ulna positive wrist, there is evidence that this increase in ulnar length during pronation leads to dorsal subluxation of the ulnar head, resulting in a decrease in ulnocarpal load and more radiocarpal load.

We found no association between linear measurements and a scaphoid fracture. This is consistent with our SSM results, were we found no association between a mode that corresponded to a linear measurement and a scaphoid fracture. However, this is in contrast to the study by Turan et al. [28], who found an association between a scaphoid fracture and all these measurements. The study by Ramos-Escalona et al. [21] reported an ulna minus as a risk factor for scaphoid fractures, but a control group was lacking, and they did not report how the measurements were performed. They hypothesize that an ulna minus is associated with a scaphoid fracture because forces passing the radial surface are higher in patients with an ulna minus than ulna plus when the wrist is in neutral position [21]. However, other studies found no association between ulnar variance and force transmission through the distal ulna [9, 32].

The precision of our SSM model differed between PA and lateral radiographs. The inter-observer and intra-observer reliability of the PA modes was mainly good and excellent. Unfortunately, most LA modes showed moderate or poor inter-observer and intra-observer reliability, which suggests our SSM model is not reliable for shape variations on LA radiographs. Our lateral radiograph protocol for SSM is simple, and we assume the quality of the radiographs and variations in the orientation of the bones to be the reason for this poor agreement of the lateral modes.

We believe the linear measurements we performed are reliable since the intra-observer reliability was excellent for all linear measurements and the inter-observer reliability was excellent for volar tilt, good for relative radial height, and moderate for radial inclination. The reliability of the lateral SSM model was poor, however, the reliability for volar tilt was excellent. This might be explained, because the landmarks used for this measurement were less influenced by the quality of the radiographs or the variation in bones.

This study has some strengths and limitations. We used SSM to explore if there is an association between a scaphoid fracture and wrist morphology. Since SSM is used to generate hypotheses and calculates independent modes of variation (shape variations), it can explore all shape variations and capture subtle differences in morphology. We used radiographs to identify morphological risk factors for a scaphoid fracture since the eventual goal of the study was to incorporate this into a prediction model, and radiographs are widely available at the emergency department. To capture the association between the more complex three-dimensional morphology of the distal radius and ulna and a scaphoid fracture, SSM could be performed on CT or MRI in the future. However, this is difficult to use in a clinical setting since CT and MRI are not standard care in these patients, which could lead to a selection bias when using these modalities for research.

Although both hospitals used standardized imaging protocols to sustain radiographs, variations in the orientation of the bones were still found. Although the reliability of our PA shape variations was good, the reliability of our lateral shape variations was poor, which could have resulted in the absence of an association between the shape variations of the wrist on the lateral radiograph and a scaphoid fracture. The low reliability of our lateral shape variation could be the result of the difficulty to obtain an exact lateral radiograph [25] which could influence our statistical shape model.

Within this study, we only assessed the distal radius and ulna, excluding the carpal bones and soft tissue structures in the wrist, which are also known to influence load distribution [9, 13]. Nevertheless, a displaced scaphoid fracture changes the shape of the scaphoid and the relation to the other carpal bones in such way that this does not represent the shape of the scaphoid as seen before trauma. Although the ligamentous structures of the wrist play a crucial role in guiding and containing carpal bone motion during overall movements of the wrist, assessing intact or ruptured wrist ligaments is not part of the standard clinical care [7]. However, previous ligamentous injury or disruption of the triangular fibrocartilage complex could influence the kinematics of the wrist leading to a different load pattern [7, 8]. Nevertheless, we excluded patients with a previous fracture of the forearm since this could influence the shape of the wrist and ulna.

In conclusion, we found a weak association between one complex wrist shape (ulna plus and narrow radius with increased volar tilt and radial inclination) on PA wrist radiographs and a scaphoid fracture. Since the association is weak and this shape might be difficult to detect on a radiograph for a physician, we believe adding this morphological risk factor to a prediction rule in the future would not enhance identifying high-risk patients for an occult scaphoid fracture.

## Supplementary Information

Below is the link to the electronic supplementary material.

Supplementary file1 (DOCX 15 KB)
