# Supplementary material for: Morphological risk factors for scaphoid fracture: a case–control study
Source: Eur J Trauma Emerg Surg. 2022 Sep 27;49(1):133–41. doi: 10.1007/s00068-022-02101-y (PMC9925522; doi:10.1007/s00068-022-02101-y)
Supplement: Supplementary file 1 — Supplementary file1 (DOCX 15 KB) [file 68_2022_2101_MOESM1_ESM.docx]

Supplementary table 1. Association shape variation of the wrist (SSM mode) and scaphoid fracture.

| Mode | adjusted Odds ratio  (95% CI) | p-value | Inter-observer ICC (95% CI) | Intra-  observer ICC  (95% CI) |
| --- | --- | --- | --- | --- |
| PA00 | 1.00 (0.74, 1.35) | 0.987 | 0.67 (0.32 – 0.85) | 0.79 (0.55 – 0.91) |
| PA01 | 0.99 (0.74, 1.33) | 0.971 | 0.92 (0.80 – 0.97) | 0.95 (0.88 – 0.98) |
| PA02 | 0.82 (0.60, 1.10) | 0.178 | 0.66 (0.23 – 0.85) | 0.89 (0.75 – 0.96) |
| PA03 | 1.00 (0.75, 1.34) | 0.980 | 0.85 (0.60 – 0.94) | 0.90 (0.76 – 0.96) |
| PA04 | **1.40 (1.04, 1.93)** | **0.031** | **0.90 (0.77 – 0.96)** | **0.89 (0.74 – 0.96)** |
| PA05 | 0.89 (0.66, 1.19) | 0.426 | 0.77 (0.51 – 0.90) | 0.87 (0.70 – 0.95) |
| PA06 | 0.81 (0.60, 1.09) | 0.167 | 0.95 (0.69 – 0.98) | 0.94 (0.86 – 0.98) |
| PA07 | 0.95 (0.71, 1.28) | 0.746 | 0.76 (0.48 – 0.90) | 0.40 (-0.04 – 0.71) |
| PA08 | 1.24 (0.92, 1.68) | 0.163 | 0.78 (0.50 – 0.91) | 0.76 (0.49 – 0.90) |
| PA09 | 1.15 (0.86, 1.56) | 0.350 | 0.81 (0.50 – 0.93) | 0.87 (0.71 – 0.95) |
| PA10 | 0.97 (0.72, 1.31) | 0.838 | 0.87 (0.50 – 0.96) | 0.75 (0.49– 0.90) |
| PA11 | 0.85 (0.62, 1.14) | 0.271 | 0.85 (0.52 – 0.95) | 0.78 (0.50 – 0.91) |
| PA12 | 1.23 (0.92, 1.67) | 0.173 | 0.79 (0.36 – 0.92) | 0.78 (0.51 – 0.91) |
| PA13 | 0.97 (0.71, 1.33) | 0.869 | 0.923 (0.83 – 0.97) | 0.89 (0.73 – 0.5) |
| PA14 | 0.98 (0.73, 1.31) | 0.876 | 0.91 (0.80 – 0.97) | 0.84 (0.65 – 0.94) |
| LA00 | 0.89 (0.67, 1.18) | 0.416 | 0.86 (0.56 – 0.95) | 0.93 (0.71 – 0.98) |
| LA01 | 1.02 (0.76, 1.38) | 0.881 | 0.56 (0.002 – 0.83) | 0.81 (0.42 – 0.93) |
| LA02 | 1.31 (0.98, 1.78) | 0.07 | 0.24 (-0.22 – 0.61) | 0.17 (-0.15 – 0.51) |
| LA03 | 1.04 (0.79, 1.38) | 0.794 | 0.38 (-0.07 – 0.70) | 0.61 (0.25 – 0.82) |
| LA04 | 0.92 (0.68, 1.23) | 0.562 | 0.42 (0.02 – 0.72) | 0.51 (0.07 – 0.78) |
| LA05 | 0.90 (0.67, 1.19) | 0.446 | 0.46 (-0.06 – 0.77) | 0.80 (0.56 – 0.91) |
| LA06 | 0.67 (0.20, 2.19) | 0.511 | 0.15 (-0.11 – 0.53) | 0.29 (-0.13 – 0.64) |
| LA07 | 0.96 (0.72, 1.28) | 0.786 | 0.28 (-0.15 – 0.63) | 0.295 (-0.11 – 0.63) |

The statistically significant mode is highlighted in bold. The odds ratio is adjusted for age and gender. The presented adjusted odds ratios per mode represent every increase in 1 standard deviation of that specific mode. The intra-class correlation coefficient (ICC) with the 95% confidence interval (CI) is reported for each mode.
